# Supplementary material for: The contribution of energy systems during 15-second sprint exercise in athletes of different sports specializations
Source: PeerJ. 2024 Aug 23;12:e17863. doi: 10.7717/peerj.17863 (PMC11348913; doi:10.7717/peerj.17863)
Supplement: Supplemental Information 1 — Abbreviations: EPCR –phosphagen system, ELA –glycolytic system, EAER –aerobic systemValues are expressed as means ± and standard deviations (p < 0.05). # significantly different from the first examination* significantly different from speed-power athletes at the same examination†significantly different from mixed athletes at the same examination§significantly different from the aerobic system¶significantly different from the glycolytic system [file peerj-12-17863-s001.docx]

Supplementary Table 1.

|  | ENDURANCE | | TEAM SPORTS | | SPEED-POWER | | Two-way ANOVA | | | | | |
| --- | --- | --- | --- | --- | --- | --- | --- | --- | --- | --- | --- | --- |
|  |  |  |  |  |  |  | Group | | Examination | | Group*Examination | |
|  | PRE | POST | PRE | POST | PRE | POST | p | η^2^ | p | η^2^ | p | η^2^ |
| Relative [%] | | | | | | | | | | | | |
| E_PCR_ | 51.8±8.1^†§¶^ | 47.1±12.8^†§^ | 33.9±14.5^§¶^ | 34.8±17.2^§¶^ | 42.3±12.3^§^ | 44.7±10.1^§^ | <0.001 | 0.335 | 0.795 | 0.001 | 0.461 | 0.032 |
| E_LA_ | 38.7±6.5^†§^ | 41.1±11.4^†§^ | 53.7±13.9^§^ | 56.9±16.5^§^ | 49.6±12.1^§^ | 48.5±9.7^§^ | <0.001 | 0.347 | 0.532 | 0.008 | 0.738 | 0.012 |
| E_AER_ | 9.5±3.2 | 11.8±5.3^*^ | 11.5±4.1 | 8.3±2.7 | 8.1±2.4 | 6.9±2.2 | <0.001 | 0.243 | 0.356 | 0.018 | 0.011 | 0.171 |
| p  (η^2^) | <0.001  (0.893) | <0.001  (0.637) | <0.001  (0.692) | <0.001  (0.683) | <0.001  (0.777) | <0.001  (0.849) |  |  |  |  |  |  |
| Absolute [kJ] | | | | | | | | | | | | |
| E_PCR_ | 37.4±16.4^§¶^ | 30.7±11.3^§^ | 26.5±17^§¶^ | 27±17^§¶^ | 34±15.6^§^ | 36.3±10.5^§^ | 0.060 | 0.112 | 0.661 | 0.004 | 0.421 | 0.036 |
| E_LA_ | 26.6±6.3^†*§^ | 26.5±6.9^†*§^ | 37.7±9.9^§^ | 38.2±8.5^§^ | 37.3±9.6^§^ | 39±9.0^§^ | <0.001 | 0.342 | 0.447 | 0.012 | 0.740 | 0.012 |
| E_AER_ | 6.4±1.7 | 7.4±3.1 | 7.7±2.5 | 5.6±1.6^#^ | 6.1±1.9 | 5.5±1.8 | 0.144 | 0.078 | 0.200 | 0.034 | 0.010 | 0.177 |
| Total EE | 70.4±19.5 | 64.7±11.5 | 72±22.6 | 70.8±19.2 | 77.3±22 | 80.8±15.7 | 0.139 | 0.080 | 0.703 | 0.003 | 0.464 | 0.032 |
| p  (η^2^) | <0.001  (0.628) | <0.001  (0.696) | <0.001  (0.552) | <0.001  (0.613) | <0.001  (0.650) | <0.001  (0.793) |  |  |  |  |  |  |
