# Supplementary material for: The contribution of energy systems during 15-second sprint exercise in athletes of different sports specializations
Source: PeerJ. 2024 Aug 23;12:e17863. doi: 10.7717/peerj.17863 (PMC11348913; doi:10.7717/peerj.17863)
Supplement: Supplemental Information 2 — Abbreviations: EPCR –phosphagen system, ELA –glycolytic system, EAER –aerobic system* significantly different from athletes with high PP and low V̇O2 at the same examination. # significantly different from athletes with high PP and high V̇O2 at the same examination. §significantly different from the aerobic system¶significantly different from the glycolytic system [file peerj-12-17863-s002.docx]

Supplementary Table 2.

|  | HIGH-V̇O_2max_  LOW-PP | | LOW-V̇O_2max_  HIGH-PP | | HIGH-V̇O_2max_  HIGH-PP | | LOW-V̇O_2max_  LOW-PP | | Two-way ANOVA | | | | | |
| --- | --- | --- | --- | --- | --- | --- | --- | --- | --- | --- | --- | --- | --- | --- |
|  |  |  |  |  |  |  |  |  | Group | | Examination | | Group*  Examination | |
|  | PRE | POST | PRE | POST | PRE | POST | PRE | POST | p | η^2^ | p | η^2^ | p | η^2^ |
| Relative [%] | | | | | | | | | | | | | | |
| E_PCR_ | 49.9±10.5^§¶^ | 45.8±10.7^§^ | 38.4±14.2^§¶^ | 40.7±12.7^§^ | 45.9±11.1^§^ | 40.8±17.4^§^ | 33.6±15.6^§¶^ | 37.2±19.4^§^ | 0.032 | 0.172 | 0.773 | 0.001 | 0.583 | 0.041 |
| E_LA_ | 39.5±9.2^§^ | 42.1±10.5^*§^ | 51.8±13^§^ | 52.3±12.2^§^ | 45.8±10.1^§^ | 49.4±15.6^§^ | 53.8±15.1^§^ | 53.8±19.7^§^ | 0.011 | 0.211 | 0.479 | 0.010 | 0.947 | 0.007 |
| E_AER_ | 10.5±2.4 | 11.2±5.9 | 8.6±3.1 | 6.8±2 | 8.2±3.3 | 9.6±3.8 | 12.5±4.6 | 8.9±2.4 | 0.002 | 0.263 | 0.294 | 0.023 | 0.132 | 0.113 |
| p  (η^2^) | <0.001  (0.817) | <0.001  (0.749) | <0.001  (0.732) | <0.001  (0.792) | <0.001  (0.813) | <0.001  (0.631) | <0.001  (0.655) | <0.001  (0.598) |  |  |  |  |  |  |
| Absolute [kJ] | | | | | | | | | | | | | | |
| E_PCR_ | 34.6±11.7^§¶^ | 31.5±12.5^§^ | 35.4±19.9^§^ | 35.5±13.6^§^ | 34.5±20.1^§^ | 27.7±13.3^§^ | 21.9±12^§¶^ | 26.3±16.2^§^ | 0.099 | 0.126 | 0.655 | 0.004 | 0.635 | 0.036 |
| E_LA_ | 26.3±5.8^*#§^ | 27.7±7.6^*#§^ | 42.4±8.3^*§^ | 43.3±6.9^*§^ | 31.8±7.4^#§^ | 31.8±8.6^#§^ | 33.5±10.6^§^ | 33.4±8.6^§^ | <0.001 | 0.447 | 0.564 | 0.007 | 0.934 | 0.009 |
| E_AER_ | 6.9±1.5 | 6.9±3.2 | 6.7±2 | 5.7±1.7 | 5.5±1.6 | 6.2±2.5 | 7.9±3.1 | 5.7±1.8 | 0.367 | 0.065 | 0.162 | 0.041 | 0.197 | 0.095 |
| Total EE | 67.8±14.2 | 66.1±17.2 | 84.7±26.3 | 84.7±14.8 | 71.9±21.5 | 65.8±9.7 | 71.9±21.5 | 65.8±9.7 | 0.002 | 0.265 | 0.635 | 0.004 | 0.825 | 0.019 |
| p  (η^2^) | <0.001  (0.713) | <0.001  (0.628) | <0.001  (0.628) | <0.001  (0.781) | <0.001  (0.550) | <0.001  (0.619) | <0.001  (0.576) | <0.001  (0.570) |  |  |  |  |  |  |
