# Supplementary material for: The contribution of energy systems during 15-second sprint exercise in athletes of different sports specializations
Source: PeerJ. 2024 Aug 23;12:e17863. doi: 10.7717/peerj.17863 (PMC11348913; doi:10.7717/peerj.17863)
Supplement: Supplemental Information 3 — Abbreviations: EPCR –phosphagen system, ELA –glycolytic system, EAER –aerobic systemValues are expressed as means ±and standard deviations (p < 0.05). # significantly different from the first examination* significantly different from athletes with low V̇O2 at the same examination§significantly different from the aerobic system¶significantly different from the glycolytic systemLegend: EPCR –phosphagen system, ELA –glycolytic system, EAER–aerobic system, EE–energy expenditure [file peerj-12-17863-s003.docx]

Supplementary Table 3.

|  | HIGH V̇O_2max_ | | LOW V̇O_2max_ | | Two-way ANOVA | | | | | |
| --- | --- | --- | --- | --- | --- | --- | --- | --- | --- | --- |
|  |  |  |  |  | Group | | Examination | | Group*Examination | |
|  | PRE | POST | PRE | POST | p | η^2^ | p | η^2^ | p | η^2^ |
| Relative [%] | | | | | | | | | | |
| E_PCR_ | 48.1±10.7^*§^ | 43.6±13.9^§^ | 36.4±14.7^§¶^ | 39.3±15.1^§¶^ | <0.009 | 0.133 | 0.757 | 0.002 | 0.163 | 0.040 |
| E_LA_ | 42.3±9.9^*§^ | 45.3±13.2^§^ | 52.6±13.6^§^ | 52.9±15.2^§^ | 0.003 | 0.163 | 0.465 | 0.011 | 0.561 | 0.007 |
| E_AER_ | 9.5±2.9 | 10.5±5 | 10.2±4.7 | 7.6±2.4 | 0.145 | 0.043 | 0.321 | 0.020 | 0.025 | 0.099 |
| p  (η^2^) | <0.001  (0.802) | <0.001  (0.669) | <0.001  (0.694) | <0.001  (0.702) |  |  |  |  |  |  |
| Absolute [kJ] | | | | | | | | | | |
| E_PCR_ | 34.5±15.5^§^ | 29.8±12.6^§^ | 30±17.9^§¶^ | 31.8±15.1^§¶^ | 0.704 | 0.003 | 0.621 | 0.005 | 0.257 | 0.026 |
| E_LA_ | 28.7±6.9^*§^ | 29.5±8.4^*§^ | 38.9±10.3^§^ | 39.3±9^§^ | <0.001 | 0.290 | 0.488 | 0.010 | 0.852 | <0.001 |
| E_AER_ | 6.3±1.6 | 6.6±2.9 | 7.2±2.5 | 5.7±1.7 | 0.999 | <0.001 | 0.172 | 0.038 | 0.054 | 0.074 |
| Total EE | 69.6±17.4 | 66±14 | 76.1±24.2 | 77±17.9 | 0.559 | 0.074 | 0.630 | 0.004 | 0.443 | 0.012 |
| p  (η^2^) | <0.001  (0.611) | <0.001  (0.610) | <0.001  (0.563) | <0.001  (0.674) |  |  |  |  |  |  |
