# Supplementary material for: The contribution of energy systems during 15-second sprint exercise in athletes of different sports specializations
Source: PeerJ. 2024 Aug 23;12:e17863. doi: 10.7717/peerj.17863 (PMC11348913; doi:10.7717/peerj.17863)
Supplement: Supplemental Information 4 — Abbreviations: EPCR –phosphagen system, ELA –glycolytic system, EAER –aerobic systemValues are expressed as means ±and standard deviations (p < 0.05).# significantly different from the first examination* significantly different from athletes with low PP at the same examination§significantly different from the aerobic system¶significantly different from the glycolytic system [file peerj-12-17863-s004.docx]

Supplementary Table 4.

|  | HIGH PEAK POWER | | LOW PEAK POWER | | Two-way ANOVA | | | | | | |
| --- | --- | --- | --- | --- | --- | --- | --- | --- | --- | --- | --- |
|  |  |  |  |  | Group | | Examination | | Group*Examination | | |
|  | PRE | POST | PRE | POST | p | η^2^ | p | η^2^ | p | η^2^ |  |
| Relative [%] | | | | | | | | | | | |
| E_PCR_ | 42.8±11.3^§^ | 40.3±14.7^§¶^ | 41.7±16.1^§^ | 42.6±15^§^ | 0.855 | <0.001 | 0.760 | 0.001 | 0.533 | 0.008 |  |
| E_LA_ | 48.1±10.8^§^ | 51.4±13.6^§^ | 46.7±14.9^§^ | 46.8±15.5^§^ | 0.347 | 0.018 | 0.464 | 0.011 | 0.470 | 0.010 |  |
| E_AER_ | 8.3±3.1^*^ | 8.1±3.2 | 11.4±3.5 | 10.1±4.8 | <0.001 | 0.235 | 0.343 | 0.186 | 0.500 | 0.009 |  |
| p  (η^2^) | <0.001  (0.784) | <0.001  (0.718) | <0.001  (0.606) | <0.001  (0.633) |  |  |  |  |  |  |  |
| Absolute [kJ] | | | | | | | | | | | |
| E_PCR_ | 36.2±18.7^§^ | 31.8±13.9^§^ | 28.3±13.8^§^ | 29.8±14^§^ | 0.132 | 0.046 | 0.621 | 0.005 | 0.308 | 0.021 |  |
| E_LA_ | 37.8±9.6^*§^ | 38.6±9.7^*§^ | 29.7±8.8^§^ | 30.3±8.3^§^ | <0.001 | 0.196 | 0.488 | 0.010 | 0.903 | 0.001 |  |
| E_AER_ | 6.2±1.9 | 5.9±2.1 | 7.3±2.3 | 6.3±2.7 | 0.106 | 0.053 | 0.186 | 0.036 | 0.467 | 0.011 |  |
| Total EE | 80.3±24.7^*^ | 76.4±16.1 | 65.4±13.9 | 66.5±16.5 | 0.005 | 0.148 | 0.629 | 0.004 | 0.387 | 0.015 |  |
| p  (η^2^) | <0.001  (0.595) | <0.001  (0.680) | <0.001  (0.543) | <0.001  (0.588) |  |  |  |  |  |  |  |
